# Blast2GO: A Comprehensive Suite for Functional Analysis in Plant Genomics

**DOI:** 10.1155/2008/619832

**Published:** 2007-04-30

**Authors:** Ana Conesa, Stefan Götz

**Affiliations:** Bioinformatics Department, Centro de Investigación Príncipe Felipe, 4012 Valencia, Spain

## Abstract

Functional annotation of novel sequence data is a primary requirement for the utilization of functional genomics approaches in plant research. In this paper, we describe the Blast2GO suite as a comprehensive bioinformatics tool for functional annotation of sequences and data mining on the resulting annotations, primarily based on the gene ontology (GO) vocabulary. Blast2GO optimizes function transfer from homologous sequences through an elaborate algorithm that considers similarity, the extension of the homology, the database of choice, the GO hierarchy, and the quality of the original annotations. The tool includes numerous functions for the visualization, management, and statistical analysis of annotation results, including gene set enrichment analysis. The application supports InterPro, enzyme codes, KEGG pathways, GO direct acyclic graphs (DAGs), and GOSlim. Blast2GO is a suitable tool for plant genomics research because of its versatility, easy installation, and friendly use.

## 1. INTRODUCTION

Functional genomics research has expanded enormously in the last decade and particularly the plant biology research community has extensively included functional genomics approaches in their recent research proposals. The number of Affymetrix plant GeneChips, for example, has doubled in the last two years [1] and extensive international genomics consortia exist for major crops (see last PAG Conference reports for an updated impression on current plant genomics, http://www.intl-pag.org). Not less importantly, many middle-sized research groups are also setting up plant EST projects and producing custom microarray platforms [2]. This massive generation of plant sequence data and rapid spread of functional genomics technologies among plant research labs has created a strong demand for bioinformatics resources adapted to vegetative species. Functional annotation of novel plant DNA sequences is probably one of the top requirements in plant functional genomics as this holds, to a great extent, the key to the biological interpretation of experimental results. Controlled vocabularies have imposed along the way as the strategy of choice for the effective annotation of the function of gene products. The use of controlled vocabularies greatly facilitates the exchange of biological knowledge and the benefit from computational resources that manage this knowledge. The gene ontology (GO, http://www.geneontology.org) [3] is probably the most extensive scheme today for the description of gene product functions but also other systems such as enzyme codes [4], KEGG pathways [5], FunCat [6], or COG [7] are widely used within molecular databases. Many bioinformatics tools and methods have been developed to assist in the assignment of functional terms to gene products (reviewed in [8]). Fewer resources, however, are available when it comes to the large-scale functional annotation of novel sequence data of nonmodel species, as would be specifically required in many plant functional genomics projects. Web-based tools for the functional annotation of new sequences include AutoFact [9], GOanna/AgBase [10], GOAnno [11], Goblet [12], GoFigure + GoDel [13], GoPET [14], Gotcha [15], HT-GO-FAT (liru.ars.usda.gov/ht-go-fat.htm), InterProScan [16], JAFA [17], OntoBlast [18], and PFP [19]. Additionally, functional annotation capabilities are usually incorporated in EST analysis pipelines. A few relevant examples are ESTExplorer, ESTIMA, ESTree. or JUICE (see [2] for a survey in EST analysis). These resources are valuable tools for the assignment of functional terms to uncharacterized sequences but usually lack high-throughput and data mining capabilities, in the first case, or provide automatic solutions without much user interactivity, in the second. In this paper, we describe the Blast2GO (B2G, www.blast2go.org) application for the functional annotation, management, and data mining of novel sequence data through the use of common controlled vocabulary schemas. The philosophy behind B2G development was the creation of an extensive, user-friendly, and research-oriented framework for large-scale function assignments. The main application domain of the tool is the functional genomics of nonmodel organisms and it is primarily intended to support research in experimental labs where bioinformatics support may not be strong. Since its release in September 2005 [20], more than 100 labs worldwide have become B2G users and the application has been referenced in over thirty peer-reviewed publications (www.blast2go.org/citations). Although B2G has a broad species application scope, the project originated in a crop genomics research environment and there is quite some accumulated experience in the use of B2G in plants, which includes maize, tobacco, citrus, Soybean, grape, or tomato. Projects range from functional assignments of ESTs [21–24] to GO term annotation of custom or commercial plant microarrays [25, 26], functional profiling studies [27–29], and functional characterization of specific plant gene families [30, 31].

In the following sections we will explain more extensively the concepts behind Blast2GO. We will describe in detail main functionalities of the application and show a use case that illustrates the applicability of B2G to plant functional genomics research.

## 2. BLAST2GO HIGHLIGHTS

Four main driving concepts form the foundation of the Blast2GO software: biology orientation, high-throughput, annotation flexibility, and data-mining capability.

*Biology orientation.* The target users of Blast2GO are biology researchers working on functional genomics projects in labs where strong bioinformatics support is not necessarily present. Therefore, the application has been conceived to be easy to install, to have minimal setup and maintenance requirements, and to offer an intuitive user interface. B2G has been implemented as a multiplatform Java desktop application made accessible by Java Webstart technology. This solution employs the higher versatility of a locally running application while assuring automatic updates provided that an internet connection is available. This implementation has proven to work very efficiently in the fast transfer to users of new functionalities and for bug fixes. Furthermore, access to data in B2G is reinforced by graphical parameters that on one hand allow the easy identification and selection of sequences at various stages of the annotation process and, on the other hand, permit the joint visualization of annotation results and highlighting of most relevant features.

*High-throughput while interactive*. Blast2GO strives to be the application of choice for the annotation of novel sequences in functional genomics projects where thousands of fragments need to be characterized. In principle, B2G accepts any amount of records within the memory resources of the user's work station. Typical data files of 20 to 30 thousand sequences can be easily annotated on a 2 Giga RAM PC (larger projects may use the graphical interface free version of Blast2GO). During the annotation process, intermediate results can be accessed and modified by the user if desired.

*Flexible annotation*. Functional annotation in Blast2GO is based on homology transfer. Within this framework, the actual annotation procedure is configurable and permits the design of different annotation strategies. Blast2GO annotation parameters include the choice of search database, the strength and number of blast results, the extension of the query-hit match, the quality of the transferred annotations, and the inclusion of motif annotation. Vocabularies supported by B2G are gene ontology terms, enzyme codes (EC), InterPro IDs, and KEGG pathways.

*Data mining on annotation results*. Blast2GO is not a mere generator of functional annotations. The application includes a wide range of statistical and graphical functions for the evaluation of the annotation procedure and the final results. Especially, (relative) abundance of functional terms can be easily assessed and visualized.

The first release of B2G covered basic application functionalities: high-throughput blast against NCBI or local databases, mapping, annotation, and gene set enrichment analysis; scalar vector graphics (SVG) combined graphs and basic distributions charts. Enhanced modules for massive blast, modification of annotation intensity, curation, additional vocabularies, high-performing customizable graphs and pathway charts, data mining and sequence handling, as well as a wide array of input and output formats have been incorporated into the Blast2GO suite.

## 3. THE BLAST2GO APPLICATION

**Figure 1:**
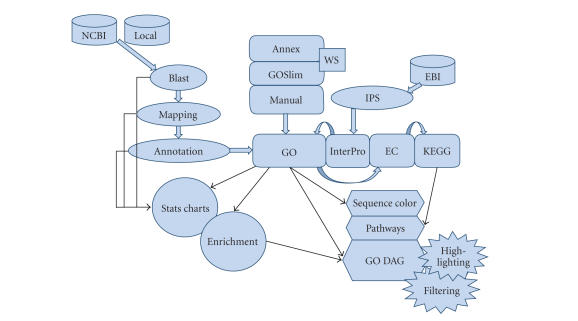
Schematic representation of Blast2GO application. GO annotations are generated through a 3-step process: blast, mapping, annotation. InterPro terms are obtained from InterProScan at EBI, converted and merged to GOs. GO annotation can be modulated from Annex, GOSlim web services and manual editing. EC and KEGG annotations are generated from GO. Visual tools include sequence color code, KEGG pathways, and GO graphs with node highlighting and filtering options. Additional annotation data-mining tools include statistical charts and gene set enrichment analysis functions.

Figure 1 shows the basic components of the Blast2GO suite. Functional assignments proceed through an elaborate annotation procedure that comprises a central strategy plus refinement functions. Next, visualization and data mining engines permit exploiting the annotation results to gain functional knowledge.

### 3.1. The annotation procedure

The Blast2GO annotation procedure consists of three main steps: blast to find homologous sequences, mapping to collect GO terms associated to blast hits, and annotation to assign trustworthy information to query sequences. Once GO terms have been gathered, additional functionalities enable processing and modification of annotation results.

*Blast step*. The first step in B2G is to find sequences similar to a query set by blast [32]. B2G accepts nucleotide and protein sequences in FASTA format and supports the four basic blast programs (blastx, blastp, blastn, and tblastx). Homology searches can be launched against public databases such as (the) NCBI nr using a query-friendly version of blast (QBlast). This is the default option and in this case, no additional installations are needed. Alternatively, blast can be run locally against a proprietary FASTA-formatted database, which requires a working www-blast installation. The Make Filtered Blast-GO-BD function in the Tools menu allows the creation of customized databases containing only GO-annotated entries, which can be used in combination with the local blast option. Other configurable parameters at the blast step are the expectation value (*e*-value) threshold, the number of retrieved hits, and the minimal alignment length (hsp length) which permits the exclusion of hits with short, low *e*-value matches from the sources of functional terms. Annotation, however, will ultimately be based on sequence similarity levels as similarity percentages are independent of database size and more intuitive than *e*-values. Blast2GO parses blast results and presents the information for each sequence in table format. Query sequence descriptions are obtained by applying a language processing algorithm to hit descriptions, which extracts informative names and avoids low-content terms such as “hypothetical protein” or “expressed protein”.

*Mapping step*. Mapping is the process of retrieving GO terms associated to the hits obtained after a blast search. B2G performs three different mappings as follows. (1) Blast result accessions are used to retrieve gene names (symbols) making use of two mapping files provided by NCBI (geneinfo, gene2accession). Identified gene names are searched in the species-specific entries of the gene product table of the GO database. (2) Blast result GI identifiers are used to retrieve UniProt IDs making use of a mapping file from PIR (Non-redundant Reference Protein database) including PSD, UniProt, Swiss-Prot, TrEMBL, RefSeq, GenPept, and PDB. (3) Blast result accessions are searched directly in the DBXRef Table of the GO database.

**Figure 2:**
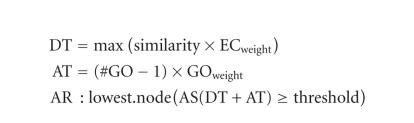
Blast2GO annotation rule.

*Annotation step*. This is the process of assigning functional terms to query sequences from the pool of GO terms gathered in the mapping step. Function assignment is based on the gene ontology vocabulary. Mapping from GO terms to enzyme codes permits the subsequent recovery of enzyme codes and KEGG pathway annotations. The B2G annotation algorithm takes into consideration the similarity between query and hit sequences, the quality of the source of GO assignments, and the structure of the GO DAG. For each query sequence and each candidate GO term, an annotation score (AS) is computed (see Figure 2). The AS is composed of two terms. The first, direct term (DT), represents the highest similarity value among the hit sequences bearing this GO term, weighted by a factor corresponding to its evidence code (EC). A GO term EC is present for every annotation in the GO database to indicate the procedure of functional assignment. ECs vary from experimental evidence, such as inferred by direct assay (IDA) to unsupervised assignments such as inferred by electronic annotation (IEA). The second term (AT) of the annotation rule introduces the possibility of abstraction into the annotation algorithm. Abstraction is defined as the annotation to a parent node when several child nodes are present in the GO candidate pool. This term multiplies the number of total GOs unified at the node by a user-defined factor or GO weight (GOw) that controls the possibility and strength of abstraction. When all ECw's are set to 1 (no EC control) and the GOw is set to 0 (no abstraction is possible), the annotation score of a given GO term equals the highest similarity value among the blast hits annotated with that term. If the ECw is smaller than one, the DT decreases and higher query-hit similarities are required to surpass the annotation threshold. If the GOw is not equal to zero, the AT becomes contributing and the annotation of a parent node is possible if multiple child nodes coexist that do not reach the annotation cutoff. Default values of B2G annotation parameters were chosen to optimize the ratio between annotation coverage and annotation accuracy [20]. Finally, the AR selects the lowest terms per branch that exceed a user-defined threshold.

The annotation step in B2G can be further adjusted by setting additional filters to the hit sequences considered as annotation source. A lower limit can be set at the e-value parameter to ensure a minimum confidence at the level of homology. Similarly, %“hit” filter has been implemented to assure that a given percentage of the hit sequence is actually spanned by the query. This parameter is of importance to prevent potential function transfer from nonmatching sequence regions of modular proteins. Additionally, the minimal hsp length required at the blast step permits control of the length of the matching region.

### 3.2. Modulation of annotation

Blast2GO includes different functionalities to complete and modify the annotations obtained through the above-defined procedure.

*Additional vocabularies*. Enzyme codes and KEGG pathway annotations are generated from the direct mapping of GO terms to their enzyme code equivalents. Additionally, Blast2GO offers InterPro searches directly from the B2G interface. The user, identified by his/her email address, has the possibility of selecting different databases available at the InterProEBI web server [33]. B2G launches sequence queries in batch, and recovers, parses, and uploads InterPro results. Furthermore, InterPro IDs can be mapped to GO terms and merged with blast-derived GO annotations to provide one integrated annotation result. In this process, B2G ensures that only the lowest term per branch remains in the final annotation set, removing possible parent-child relationships originating from the merging action.

*Annotation fine-tuning*. Blast2GO incorporates three additional functionalities for the refinement of annotation results. Firstly, the Annex function allows annotation augmentation through the Second Layer concept developed by The Norwegian University of Science and Technology (http://www.goat.no, [34]). Basically, the Second Layer database is a collection of manually curated univocal relationships between GO terms from the different GO categories that permits the inference of biological process and cellular component terms from molecular function annotations. Up to 15% of annotation increase and around 30% of GO term confirmations are obtained through the Annex dataset [20]. Secondly, annotation results can be summarized through GOSlim mapping. GOSlim consists of a subset of the gene ontology vocabulary encompassing key ontological terms and a mapping function between the full GO and the GOSlim. Different GOSlim mappings are available, adapted to specific biological domains. At present, GOSlim mappings for plant, yeast, from GOA and Tair, as well as a generic one are available from the GO through Blast2GO. Thirdly, the manual curation function means that the user has the possibility of editing annotation results and manually modifying GO terms and sequence descriptors.

### 3.3. Visualization and data mining

One aspect of the uniqueness of the Blast2GO software is the availability of a wide array of functions to monitor, evaluate, and visualize the annotation process and results. The purpose of these functions is to help understand how functional annotation proceeds and to optimize performance.

*Statistical charts*. Summary statistics charts are generated after each of the annotation steps. Distribution plots for e-value and similarity within blast results give an idea of the degree of homology that query sequences have in the searched database. Once mapping has been completed, the user can check the distribution of evidence codes in the recovered GO terms and the original database sources of annotations. These charts give an indication of suitable values for B2G annotation parameters. For example, when a good overall level of sequence similarity is obtained for the dataset, the default annotation cutoff value could be raised to improve annotation accuracy. Similarly, if evidence code charts indicate a low representation of experimentally derived GOs, the user might choose to increase the weight given to electronic annotations. After the final annotation step, new charts show the distribution of annotated sequences, the number of GOs per sequence, the number of sequences per GO, and the distribution of annotations per GO level, which jointly provide a general overview of the performance of the annotation procedure.

**Figure 3:**
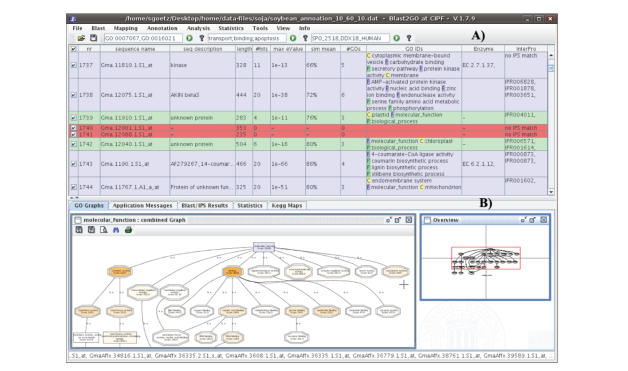
Blast2GO user interface. (A) Main sequence table showing sequence color codes. (B) Graphical tab showing a combined graph with score highlighting.

*Sequence coloring*. The visual approach of B2G is further represented by the color code given to annotated sequences. During the annotation process, the background color of active sequences changes according to their analysis status. Nonblasted sequences are displayed in white and change to light red once a positive blast result is obtained. If the result was negative, they will stay dark red. Mapped sequences are depicted in green while annotated sequences become blue. Finally, manually curated sequences can be labeled and colored purple (see Figure 3(A)). Sequence coloring is a simple and effective way of identifying sequences that have reached differential stages during the annotation process. Furthermore, sequences can be selected by their color. This is a very useful function for the interactive use of the application. For example, sequences that stayed dark red after blast (no positive result) can be selected to be launched to InterProScan. Sequences that remained green (mapping code) after the annotation step can be selected and reannotated with more permissive parameters.

*Combined graph*. A core functionality of Blast2GO is the joint visualization of groups of GO terms within the structure of the GO DAG. The combined graph function is typically used to study the collective biological meaning of a set of sequences. Combined graphs are a good alternative to enrichment analysis (see below) where no reference set is to be considered or the number of involved sequences is low. B2G includes several parameters to make these combined graphs easy to analyze and navigate. Firstly, the ZWF format [35], a powerful scalable vector graphics engine, has been adopted to make zooming and browsing through the DAG fast and light. Secondly, annotation-rich areas of the generated DAG can be readily spotted by a node-coloring function. B2G colors nodes either by the number of sequences gathered at that term (additive function) or by a node information score (exponential function, ∑_GOs_seq⋅*α* ^dist^) that considers the places of direct annotation. This B2G score takes into account the amount of sequences collected at a given term but penalizes by the distance to the node of actual annotation [20]. The B2G score has shown to be a useful parameter for the identification of “hot” terms within a specific DAG (see Figure 3(B); Conesa, unpublished). Thirdly, the extension and density of the plotted DAG can be modulated by a node filter function. When the number of sequences involved in the combined graph is large, the resulting DAG can be too big to be practical. B2G permits filtering out of low informative terms by imposing a threshold on the number of annotated sequences or B2G scores for a node to be displayed. In this case, the number of omitted nodes is given for each branch, which is an indication of the level of local compression applied to that branch.

*Enrichment analysis*. A typical data mining approach applied in functional genomics research is the identification of functional classes that statistically differ between two lists of terms. For example, one might want to know the functional categories that are over- or underrepresented in the set of differentially expressed genes of a microarray experiment, or it could be of interest to find which functions are distinctly represented between different libraries of an EST collection. Blast2GO has integrated the Gossip [36] package for statistical assessment of differences in GO term abundance between two sets of sequences. This package employs the Fisher's exact test and corrects for multiple testing. For this analysis, the involved sequences with their annotations must be loaded in the application. B2G returns the GO terms under- or overrepresented at a specified significance value. Results are given as a plain table and graphically as a bar chart and as a DAG with nodes colored by their significance value. Also in this case, graph pruning and summarizing functions are available.

### 3.4. Other functionalities

Next to the annotation and data mining functions, Blast2GO comprises a number of additional functionalities to handle data. In this section, we briefly comment on some of them.

*Import and export*. B2G provides different formats for the exchange of data. Typically, B2G inputs are FASTA-formatted sequences and returns a tab-delimited file with GO annotations. Other supported output formats are GOstats and GOSpring. Furthermore, B2G also accepts blast results in xml format. This option permits skipping the first step of the B2G annotation procedure when a blast result is already present. Similarly, when accession IDs or gene symbols are known for the query sequences, these can be directly uploaded in B2G and the application will query the B2G database for their annotations. Moreover, the Main Sequence table (see Figure 3) can be saved to a file at any moment to store intermediate results. Finally, graph and enrichment analysis results are presented both graphically and as text files.

*Validation*. The “true path rule” defined by the gene ontology consortium for the GO DAG assures that all the terms in the pathway from a term up to the root must always be true for a given gene product. The B2G annotation validation function applies this property to annotation results by removing any parent term that has a child within the sequence annotation set. B2G always executes validation after any modification has been made to the existing annotation, for example, after InterPro merging, Annex augmentation, or manual curation.

*Comparison of two sets of GO terms*. Given two annotation results, Blast2GO can compare their implicit DAG structures. B2G computes the number of identical nodes, more general and more specific terms within the same branch, and terms located to different branches or different GO main categories. Comparison is directional; this means that the active annotation file is contrasted to a reference or external one. Each GO term is compared to all terms in the reference set and the best matching comparison result is recorded. Once a term is matched, it is removed from the query set.

### 3.5. Some performance figures

The annotation accuracy of Blast2GO has been evaluated by comparing B2G GO annotation results to the existing annotation in a set of manually annotated Arabidopsis proteins that had been previously removed from the nr database. This evaluation indicated that using B2G default parameters, nearly 70% of identical branch recovery was achievable, which is at the top end of the methods that are based on homology search [20]. More recent evaluations have shown that Blast2GO annotation behavior is consistent across species and datasets. In general, the blast step has shown to be decisive in the annotation coverage. For a great deal of sequences with a positive blast result, functional information is available in the GO database and the final annotation success is related to the length and quality of the query sequence and the strictness of annotation parameters. Typically and using default parameters, around 50–60% of annotation success is common for EST datasets and slightly higher values are obtained for full-length proteins (Table 1).

On average, between 3 and 6 GO terms are assigned per sequence at a mean GO level very close to 5. InterPro, Annex, and GOw annotation parameters significantly increase annotation intensity—around 15%—and validate annotation results. Furthermore, default annotation options tend to provide coherent results and resemble the functional assignment obtained by a human computational reviewed analysis [37].

### 3.6. Use case

In this section, we present a typical use case of Blast2GO to illustrate the major application features described in the previous sections. We will address the functional annotation of the Soybean Affymetrix GeneChip. The GeneChip Soybean Genome Array targets over 37,500 Soybean transcripts (www.affymetrix.com). The array also contains transcripts for studying two pathogens important for Soybean research. Sequence data and a detailed annotation sheet for the Soybean Genome Array are provided at the Blast2GO site (http://blast2go.bioinfo.cipf.es/b2gdata/soybean).

**Blast**

**Figure 4:**
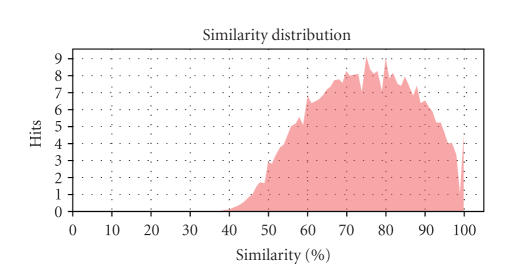
Similarity distribution of Soybean GeneChip. Similarity is computed of each query-hot pair as the sum of similarity values for all matching hsps.

**Figure 5:**
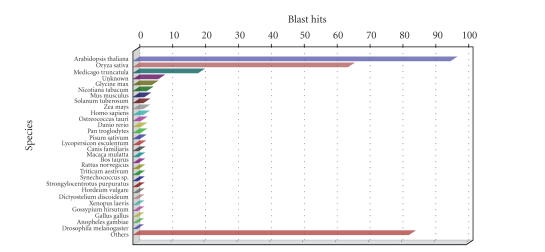
Species distribution chart of Soybean GeneChip after blastx to NCBI nr.

Sequence data in FASTA format were uploaded into the application from the menu File → Open File. After selecting the Blast menu, a dialog opens where we can indicate the parameters for the blast step. In our case, the easiest option is to select the nr protein database and perform blast remotely on the NCBI server through Qblast. Additional blast parameters are kept at default values: *e*-value threshold of 1*e*-3 and a recovery of 20 hits per sequence. These permissive values are chosen to retrieve a large amount of information at this first time-consuming step. Annotation stringency will be decided later in the annotation procedure. Furthermore, we set the hsp filter to 33 to avoid hits where the length of the matching region is smaller than 100 nucleotides. After launching, blast sequences turn red as results arrive, up to a total of 22,788. Once blast is completed, we can visualize different charts (similarity, *e*-value, and species distributions, see supplementary material available online at doi:10.1155/2008/619832) to get an impression of the quality of the query sequences and the blast procedure. For example, Statistics → Blast statistics → Similarity distribution chart (see Figure 4) shows that most sequences have blast similarity values of 50–60% or higher. This information is useful for choosing the annotation cutoff parameter at the annotation step, and suggests that taking a value of 60 would be adequate. Furthermore, the Species distribution chart (see Figure 5) shows a great majority of Arabidopsis sequences within the blast hits, followed by Cotton, Medicago, Glycine, and Nicotiana.

**Mapping**

**Figure 6:**
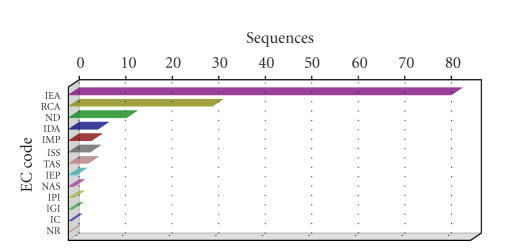
Evidence code distribution chart of Soybean GeneChip after mapping to B2G database.

Mapping is a nonconfigurable option launched from the menu Mapping → Make Mapping. GO terms could be found for 21,079 sequences (56%). Mapping charts (menu Statistics → Mapping Statistics) permit the evaluation of mapping results. The evidence code distribution chart (see Figure 6) shows an overrepresentation of electronic annotations, although other nonautomatic codes, such as review by computational analysis (RCA), inferred by mutant phenotype (IMP), or inferred by direct assay (IDA) are also well represented. This suggests that an annotation strategy that promotes nonelectronic ECs would be meaningful as it would benefit from the high-quality GO terms without totally excluding electronic annotations. Therefore, the default EC weights (menu Annotation → Set Evidence Code Weights) that adjust proportionally to the reliability of the source annotation will be maintained at the annotation step.

**Annotation**

**Figure 7:**
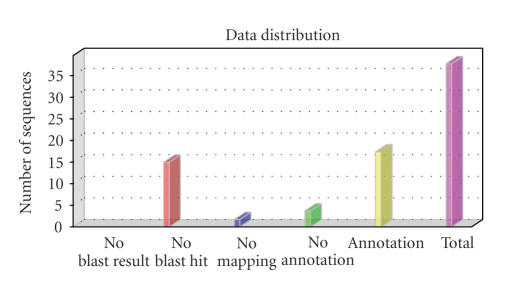
Annotation process results for Soybean Affymetrix GeneChip.

**Figure 8:**
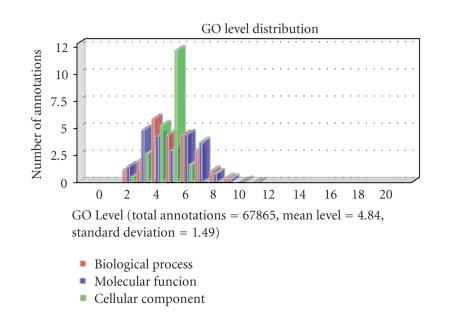
GO level distribution chart for Soybean Affymetrix GeneChip. Most sequences have between 3 and 6 GO terms annotated.

Taking into consideration the charts generated by the previous steps, we have chosen an annotation configuration with an *e*-value filter of 1*e*-6, default gradual EC weights, a GO weight of 15, and an annotation cutoff of 60. This implies that only sequences with a blast *e*-value lower than 1*e*-6 will be considered in the annotation formula, that the query-hit similarity value adjusted by the EC weight of the GO term should be at least 60, and that abstraction is strongly promoted. This annotation configuration resulted in 17,778 successfully GO annotated sequences with a total of 70,035 GO terms at a mean GO level (distance of the GO term to the ontology root term) of 4.72. Furthermore, 6,345 enzyme codes were mapped to a total of 5,390 sequences. Once annotation has been completed, we can visualize the results at each step of the annotation process (see Figure 7). Reannotation is possible by selecting green or red sequences (Tools → Select Sequences by Color) and rerunning blast, mapping, and annotation with different, more permissive parameters. In this way, we obtain a trustworthy annotation for most sequences and behave more permissively only for those sequences which are hard to annotate. Other charts available at the Annotation Statistics menu show the distribution of GO levels (see Figure 8), the length of annotated sequences, and the histogram of GO term abundance.

**Annotation augmentation**

**Figure 9:**
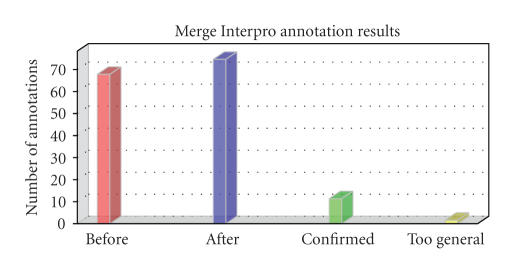
InterPro merging statistics. Red column: total number of GO annotations before adding InterPro-based GO terms. Blue: total number of GO annotations after adding InterPro-based GO terms. Green: number of blast-based GO terms confirmed after InterPro merging. Yellow: too general terms removed after InterPro merging.

Blast-based GO annotations can be increased by means of the integrated InterProScan function available under Annotation → Run InterProScan. The user must provide his/her email address and select the motif databases of interest. An InterProScan search against all EBI databases resulted in the recovery of motif functional information for 11,347 sequences and a total of 8,046 GO terms. Once merged to the already existing annotation (Annotation → Add InterProScan GOs to Annotation), 1,189 additional sequences were annotated (see Figure 9). Once Blast plus InterProScan annotations have been gathered, a useful step is to complete implicit annotations through the Annex function (Annotation → Augment Annotation by Annex). After this step, it is recommended to run the function to remove first-level annotations (under Annotation menu). In our use case, the Annex function resulted in the addition of 8,125 new GO terms and a confirmation of 3,892 annotations, which is an average contribution of the Annex function [37].

**Manual curation**

The manual annotation tool is a useful functionality when information on the automatically generated annotation needs to be changed. For example, the target of GmaAffx.69219.1.S1_at probe was found to be the UDP-glycosyltransferase. The automatic procedure assigned GO terms metabolic process (GO:0008152) and transferase activity, transferring hexosyl groups (GO:0016758) to this sequence. However, as we are aware of the ER localization of this enzyme and its involvement in protein maturation, we would like to add this information to the existing annotation. The manual curation function is available at the Sequence Menu which is displayed by mouse right button click on the selected sequence. From this Menu, the blast and annotation results for this particular sequence can be visualized. Selection of Change Annotations and Description edits the annotation record of GmaAffx.69219.1.S1_at. We can now type in the Annotations box the terms GO:0005783 (endoplasmic reticulum) and GO:0006464 (protein modification process) and mark the manual annotation box. The new annotations are then added and the sequence turns purple (manual annotation color code).

**GOSlim**

As the number of sequences and different GO terms in the Soybean array is quite large, we are interested in a simpler representation of the functional content of the data. An appropriate option is to map annotations into a GOSlim. At Annotation → Change to GOSlim View, we can select an appropriate GOSlim (generic_plant) for this dataset. Upon completion of slimming sequences acquire the yellow GOSlim coding. The original annotations are stored and can be recovered at any moment. GOSlim mapping generated a set of 105 different annotating GO terms on 18,820 sequences with a mean GO level of 3.41. This means around 40 times less functional diversity than in the original annotation (4533 different terms) and an increase of almost 2 levels of the mean annotation depth.

**Combined graph**

**Figure 10:**
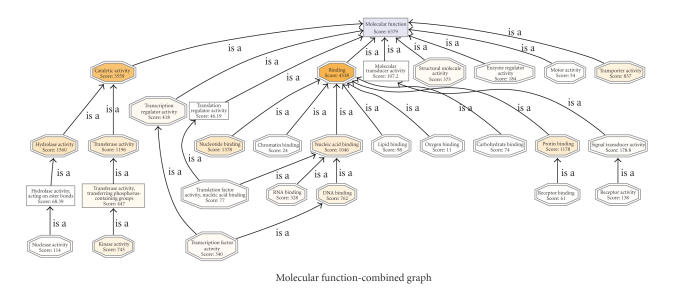
Molecular function combined graph of GOSlim annotation of the Soybean Affymetrix GeneChip. Nodes are colored by score value.

Once the slimmed annotation is obtained, we can visualize the functional information of the Soybean Genome Array on the GO DAG. This functionality is available under Analysis → Combined Graph. At the Dialog we must indicate the GO category to display (e.g., biological process). To obtain a compact representation of the information, two filters can be applied. For example, by setting the sequence filter to 20, only those nodes with at least 20 sequence assignments will be displayed. By setting the score filter to 20, additionally, parent nodes that do not annotate more sequences than their children terms will be omitted from the graph. Node coloring by score value highlights the areas in the resulting DAG where sequence annotations are most concentrated. Figure 10 shows the Combined Graph for the Molecular Function Category. The two most intensively colored terms at the second GO level indicate the two most abundant functional categories in the Soybean Chip: catalytic activity and binding. Highlighting at lower levels reveals other, most informative, highly represented functional terms, such as hydrolase activity (level 3), kinase activity (level 4), transcription factor activity (level 3), protein binding (level 3), nucleotide binding (level 3), and transporter activity (level 2). The reader is referred to the annotation sheet URL (http://blast2go.bioinfo.cipf.es/b2gdata/soybean) for figure navigation.

**Enrichment analysis**

**Figure 11:**
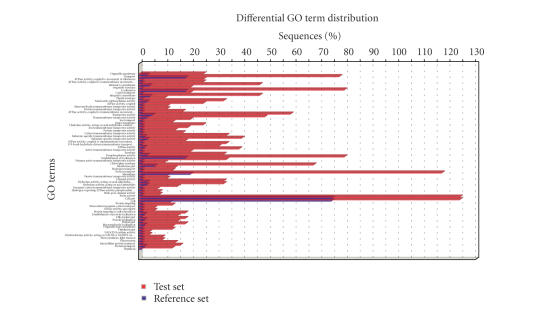
Bar chart for functional category enrichment analysis of Soybean membrane proteins. The *Y*-axis shows significantly enriched GO terms and the *X*-axis give the relative frequency of the term. Red bars correspond to test set (membrane) and blue bars correspond to the whole Soybean genome array.

**Figure 12:**
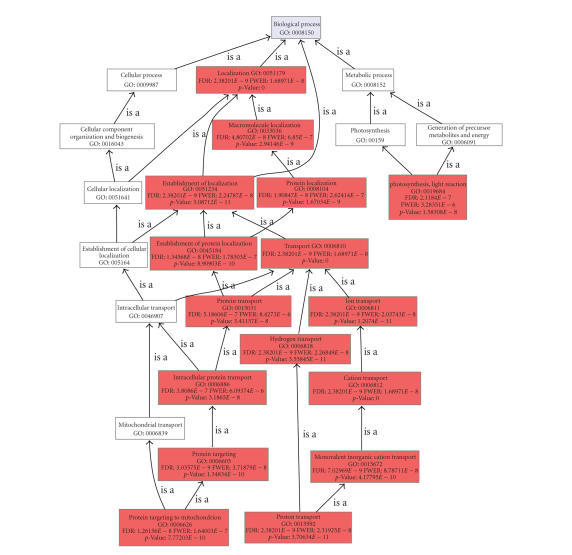
Enriched graph (biological process) of the Soybean membrane subset of sequences. Node filter has been set at FDR < 1*e*-6. Nodes are colored accordingly to their FDR value in the Fisher exact's test against the whole Soybean genome array.

The enrichment analysis function in B2G executes a statistical assessment of differences in functional classes between two groups of sequences. To illustrate this function, we have selected all sequences in the Soybean chip which contain the word “membrane” within their description—132 sequences—and compared their annotations to the whole chip. We go to Analysis → Enrichment Analysis → Make Fisher's Exact Test and browse for a text file containing the test set with the names of membrane sequences. As the comparison is made against the complete microarray dataset loaded into the application, no file needs to be selected as Reference. We uncheck the two-tail box to perform only positive enrichment analysis. Upon completion a table with test statistical results is presented in the Statistics tab. This table contains significant GO terms which are ranked according to their significance. Three different significance parameters are given for false-positive control: false discovery rate (FDR), family-wise error rate (FWER), and single test *P*-value (Fisher *P*-value) (see [36] for details). By taking a FDR significance threshold of 0.05, we obtain those functionalities that are strongly significant for membrane proteins in the Soybean Chip. These refer to processes related to transport, protein targeting, and photosynthesis as might be expected for a plant species. Graphical representations of these results can be generated at Analysis → Enrichment Analysis → Bar Chart and Analysis → Enrichment Analysis → Make Enriched Graph. The Bar Chart shows, for each significant GO term, frequency differences between the membrane and the whole chip datasets (see Figure 11). The Enriched Graph shows the DAG of significant terms with a node-coloring proportional to the significance value. This representation helps in understanding the biological context of functional differences and to find pseudoredundancies in the results—parent-child relationships within significant terms—(see Figure 12).

**Export results**

Once different analyses have been completed the data can be exported in many different ways. The annotation format (menu File → Export → Export Annotations) is the default format for export/import in B2G and simply consists of a tab-delimited file with two columns, one for sequences and other for annotation IDs. Another useful export format is GeneSpring, for communication with this interesting application, which consists of one row per sequence and three different columns showing the descriptions of the GO terms at the three main GO categories. Graphs can be saved in png format. Additionally, all information contained in the Combined Graph can be generated as table (including sequences, GO IDs, levels, and scores) and exported (Analysis → Export Graph Information).

The analysis presented in this use case took about 15 days to complete. Four days were necessary to obtain the totality of 37,500 blast results from the NCBI while twelve days were required for the InterProScan at the EBI web server. Mapping and Annotation were ready within a few hours and one day was necessary to collect and evaluate charts. This shows that with the adequate tools and some training, functional annotation of a plant genome-wide sequence collection is in reach within a couple of weeks.

## 4. CONCLUSIONS

Functional annotation of novel sequence data is a key requirement for the successful generation of functional genomics in biological research. The Blast2GO suite has been developed to be a useful support to these approaches, especially (but not exclusively) in nonmodel species. This bioinformatics tool is ideal for plant functional genomics research because of the following: (1) it is suitable for any species but can be also customized for specific needs, (2) it combines high throughput with interactivity and curation, and (3) it is user-friendly and requires low bioinformatics efforts to get it running. In our opinion, the major B2G strength is the combination of functional annotation and data mining on annotation results, which means that, within one tool, researchers can generate functional annotation and assess the functional meaning of their experimental results. Further developments of Blast2GO will reinforce this second aspect thought the integration of the tool with the Babelomics (www.babelomics.org, [38]) and GEPAS suites (www.gepas.org, [39]) for the statistical analysis of functional profiling data.

## Figures and Tables

**Table 1:** Blast2GO performance figures of seven cDNA datasets. FE: percentage of sequences with some functional evidence (Mapping or InterProScan positive). BA: percentage of blast-based annotated sequences, #GO: number of GOs per sequence. GO *L*: mean GO level. IP: percentage of annotation increase by InterProScan. Ann: percentage of annotation increase by Annex. TA: total percentage of annotated sequences (including blast and InterPro). Datasets are described in [37].

DataSet	FE	BA	no. GOs	GO *L*	IP	Ann	TA
*C. clementina*	70.2	58.2	4.4	5.10	7.9	11.8	62.3
*M. incog*	70.7	55.7	5.6	4.95	11.8	9.9	63.9
*T. harzianum*	61.1	47.7	3.6	5.27	14.4	16.2	53.4
*G. max*	61.8	51.1	4.3	5.11	6.1	11.8	53.5
*P. flesus*	50.1	34.4	5.2	5.07	21.9	10.6	45.1
*A. phagocytophilum*	56.6	42.5	3.0	4.91	35.4	20.9	49.1
Whale metagenome	69.5	50.7	3.0	4.45	17.6	18	58.8

## References

[1] Conesa A, Forment J, Gadea J, van Dijk J, Falciani F (2007). Microarray technology in agricultural research. *Microarray Technology Through Applications*.

[2] Nagaraj SH, Gasser RB, Ranganathan S (2007). A hitchhiker's guide to expressed sequence tag (EST) analysis. *Briefings in Bioinformatics*.

[3] Ashburner M, Ball CA, Blake JA (2000). Gene Ontology: tool for the unification of biology. The Gene Ontology Consortium. *Nature Genetics*.

[4] Schomburg I, Chang A, Ebeling C (2004). BRENDA, the enzyme database: updates and major new developments. *Nucleic Acids Research*.

[5] Ogata H, Goto S, Sato K, Fujibuchi W, Bono H, Kanehisa M (1999). KEGG: Kyoto Encyclopedia of Genes and Genomes. *Nucleic Acids Research*.

[6] Ruepp A, Zollner A, Maier D (2004). The FunCat, a functional annotation scheme for systematic classification of proteins from whole genomes. *Nucleic Acids Research*.

[7] Tatusov RL, Fedorova ND, Jackson JD (2003). The COG database: an updated version includes eukaryotes. *BMC Bioinformatics*.

[8] Kumar S, Dudley J (2007). Bioinformatics software for biologists in the genomics era. *Bioinformatics*.

[9] Koski LB, Gray MW, Lang BF, Burger G (2005). AutoFACT: an automatic functional annotation and classification tool. *BMC Bioinformatics*.

[10] McCarthy FM, Bridges SM, Wang N (2007). AgBase: a unified resource for functional analysis in agriculture. *Nucleic Acids Research*.

[11] Chalmel F, Lardenois A, Thompson JD (2005). GOAnno: GO annotation based on multiple alignment. *Bioinformatics*.

[12] Groth D, Lehrach H, Hennig S (2004). GOblet: a platform for Gene Ontology annotation of anonymous sequence data. *Nucleic Acids Research*.

[13] Khan S, Situ G, Decker K, Schmidt CJ (2003). GoFigure: automated Gene Ontology annotation. *Bioinformatics*.

[14] Vinayagam A, del Val C, Schubert F (2006). GOPET: a tool for automated predictions of Gene Ontology terms. *BMC Bioinformatics*.

[15] Martin DMA, Berriman M, Barton GJ (2004). GOtcha: a new method for prediction of protein function assessed by the annotation of seven genomes. *BMC Bioinformatics*.

[16] Zdobnov EM, Apweiler R (2001). InterProScan—an integration platform for the signature-recognition methods in InterPro. *Bioinformatics*.

[17] Friedberg I, Harder T, Godzik A (2006). JAFA: a protein function annotation meta-server. *Nucleic Acids Research*.

[18] Zehetner G (2003). OntoBlast function: from sequence similarities directly to potential functional annotations by ontology terms. *Nucleic Acids Research*.

[19] Hawkins T, Luban S, Kihara D (2006). Enhanced automated function prediction using distantly related sequences and contextual association by PFP. *Protein Science*.

[20] Conesa A, Götz S, García-Gómez JM, Terol J, Talón M, Robles M (2005). Blast2GO: a universal tool for annotation, visualization and analysis in functional genomics research. *Bioinformatics*.

[21] Terol J, Conesa A, Colmenero JM (2007). Analysis of 13000 unique *Citrus* clusters associated with fruit quality, production and salinity tolerance. *BMC Genomics*.

[22] Vizcaíno JA, González FJ, Suárez MB (2006). Generation, annotation and analysis of ESTs from *Trichoderma harzianum* CECT 2413. *BMC Genomics*.

[23] Faria-Campos AC, Moratelli FS, Mendes IK (2006). Production of full-length cDNA sequences by sequencing and analysis of expressed sequence tags from 
*Schistosoma mansoni*. *Memorias do Instituto Oswaldo Cruz*.

[24] Durica DS, Kupfer D, Najar F (2006). EST library sequencing of genes expressed during early limb regeneration in the fiddler crab and transcriptional responses to ecdysteroid exposure in limb bud explants. *Integrative and Comparative Biology*.

[25] Williams TD, Diab AM, George SG (2006). Development of the GENIPOL European flounder (*Platichthys flesus*) microarray and determination of temporal transcriptional responses to cadmium at low dose. *Environmental Science and Technology*.

[26] Gandía M, Conesa A, Ancillo G (2007). Transcriptional response of *Citrus aurantifolia* to infection by *Citrus tristeza virus*. *Virology*.

[27] Reyes-Prieto A, Hackett JD, Soares MB, Bonaldo MF, Bhattacharya D (2006). Cyanobacterial contribution to algal nuclear genomes is primarily limited to plastid functions. *Current Biology*.

[28] Nueda MJ, Conesa A, Westerhuis JA (2007). Discovering gene expression patterns in time course microarray experiments by ANOVA-SCA. *Bioinformatics*.

[29] Williams TD, Diab AM, George SG, Sabine V, Chipman JK (2007). Gene expression responses of European flounder (*Platichthys flesus*) 
to 17-*β* estradiol. *Toxicology Letters*.

[30] Ma J, Morrow DJ, Fernandes J, Walbot V (2006). Comparative profiling of the sense and antisense transcriptome of maize lines. *Genome Biology*.

[31] Nelson RT, Shoemaker R (2006). Identification and analysis of gene families from the duplicated genome of soybean using EST sequences. *BMC Genomics*.

[32] Altschul SF, Gish W, Miller W, Myers EW, Lipman DJ (1990). Basic local alignment search tool. *Journal of Molecular Biology*.

[33] Labarga A, Valentin F, Anderson M, Lopez R (2007). Web services at the European bioinformatics institute. *Nucleic Acids Research*.

[34] Myhre S, Tveit H, Mollestad T, Lægreid A (2006). Additional Gene Ontology structure for improved biological reasoning. *Bioinformatics*.

[35] Pietriga E A toolkit for addressing HCI issues in visual language environments.

[36] Blüthgen N, Brand K, Cajavec B, Swat M, Herzel H, Beule D (2005). Biological profiling of gene groups utilizing Gene Ontology. *Genome Informatics*.

[37] Goetz S, García-Gómez JM, Terol J High throughput functional annotation and data mining with the Blast2GO suite.

[38] Al-Shahrour F, Minguez P, Tárraga J (2006). BABELOMICS: a systems biology perspective in the functional annotation of genome-scale experiments. *Nucleic Acids Research*.

[39] Montaner D, Tárraga J, Huerta-Cepas J (2006). Next station in microarray data analysis: GEPAS. *Nucleic Acids Research*.

